# An Underwater Source Location Privacy Protection Scheme Based on Game Theory in a Multi-Attacker Cooperation Scenario

**DOI:** 10.3390/s24092851

**Published:** 2024-04-30

**Authors:** Beibei Wang, Xiufang Yue, Kun Hao, Yonglei Liu, Zhisheng Li, Xiaofang Zhao

**Affiliations:** 1 School of Control and Mechanical Engineering, Tianjin Chengjian University, Tianjin 300384, China; wbbking@163.com; 2 School of Computer and Information Engineering, Tianjin Chengjian University, Tianjin 300384, China; xiufangyue@163.com (X.Y.); yongleiliu@vip.163.com (Y.L.); lzs@tcu.edu.cn (Z.L.); zhaoxftju@tju.edu.cn (X.Z.)

**Keywords:** underwater acoustic sensor networks, source location privacy, passive attacks, virtual coordinate system, network coding, evolutionary game theory

## Abstract

Ensuring source location privacy is crucial for the security of underwater acoustic sensor networks amid the growing use of marine environmental monitoring. However, the traditional source location privacy scheme overlooks multi-attacker cooperation strategies and also has the problem of high communication overhead. This paper addresses the aforementioned limitations by proposing an underwater source location privacy protection scheme based on game theory under the scenario of multiple cooperating attackers (SLP-MACGT). First, a transformation method of a virtual coordinate system is proposed to conceal the real position of nodes to a certain extent. Second, through using the relay node selection strategy, the diversity of transmission paths is increased, passive attacks by adversaries are resisted, and the privacy of source nodes is protected. Additionally, a secure data transmission technique utilizing fountain codes is employed to resist active attacks by adversaries, ensuring data integrity and enhancing data transmission stability. Finally, Nash equilibrium could be achieved after the multi-round evolutionary game theory of source node and multiple attackers adopting their respective strategies. Simulation experiments and performance evaluation verify the effectiveness and reliability of SLP-MACGT regarding aspects of the packet forwarding success rate, security time, delay and energy consumption: the packet delivery rate average increases by 30%, security time is extended by at least 85%, and the delay is reduced by at least 90% compared with SSLP, PP-LSPP, and MRGSLP.

## 1. Introduction

The ocean, as a new frontier of overall national security, plays a more prominent role in safeguarding national sovereignty, security, and development interests. More and more countries pay attention to the monitoring of the marine environment by utilizing underwater acoustic sensor networks (UASNs) for scientific exploration, commercial development, military applications, etc. [1,2]. Source location privacy protection in UASNs is at the forefront of contemporary research due to its critical role in ensuring the security and integrity of underwater communication systems [3]. These networks consist of a large number of underwater sensor nodes that collect and transmit critical information. However, the precise location of these sensor nodes is sensitive information, and its exposure may lead to security threats such as adversarial tracking, resource targeting, and privacy breaches [4]. Therefore, ensuring reliable source location privacy protection in UASNs is critical [5].

When protecting source location privacy in UASNs, traditional encryption and authentication techniques usually ignore the strategic interaction and competition between network nodes, which may lead to failure to consider the strategic actions that attackers may take, thus reducing the effectiveness of privacy protection [6]. In addition, UASNs employ acoustic communication [7,8], and the underwater environment presents unique challenges such as limited bandwidth, high propagation latency, and dynamic network topologies, which require innovative and customized source location privacy protection schemes, rather than simply applying technologies from terrestrial networks to underwater environments. In this particular context, solutions must be developed for the needs and limitations of underwater acoustic sensor networks to ensure that privacy protection does not sacrifice network performance and availability. Hence, developing mechanisms based on methods such as game theory to account for competition and cooperation between nodes is essential for increasing the level of source location privacy [9,10].

In regard to source location privacy protection in UASNs, it is necessary to consider three main problems: source location privacy, latency and energy [11]. Among them, safeguarding the location privacy of source nodes is the paramount issue in UASNs. In underwater acoustic sensor networks, attackers can deduce a source node’s location by analyzing acoustic signals [12]. Therefore, it is necessary to adopt privacy protection mechanisms, such as false source technology and multi-path routing, to reduce the possibility of attackers obtaining location information [13]. However, these mechanisms introduce problems of latency and energy consumption. Latency is another key factor to be balanced in UASNs. Excessive communication latency is unacceptable in real-time or interactive applications. Introducing source location privacy protection mechanisms may increase communication latency as additional processing steps take time. Therefore, the relationship between location privacy protection and communication latency needs to be balanced to meet the latency requirements of specific applications. Sensor nodes in UASNs usually rely on limited battery power [14]. Balancing between energy efficiency and location privacy protection is essential because location privacy protection mechanisms could introduce additional energy overhead in UASNs [15].

In this paper, a source location privacy protection scheme (SLP-MACGT) based on game theory is proposed to protect the location privacy of sensor nodes in multi-attacker cooperation scenarios by exploring the application of source location privacy challenges in depth. Through this study, a new perspective and solution for source location privacy protection in underwater acoustic networks are provided to meet the growing security needs. The main contributions of this paper are summarized as follows:

- Establish a multi-attacker model. Considering the cooperative behavior among attackers, attackers can launch active attacks in addition to common passive attacks. In this paper, we use game theory to analyze the cooperation and competition between multiple attackers so as to design a comprehensive defense strategy against multiple threats;
- A virtual coordinate system transformation method is proposed as a means to protect the location privacy of source nodes. The real location information of the source node is effectively hidden to reduce the success probability of potential attackers;
- A new relay node selection strategy is proposed. The number of hops between the source node and the target node is increased to confuse the attacker’s inference about the source node location, thus reducing the possibility that the attacker can obtain the location of the source node by monitoring network traffic and strengthening the network’s defense against passive attacks. Furthermore, a secure data transmission method based on fountain codes is proposed to resist the active attack of attackers. By introducing redundant information, the reliability of data transmission is improved, the feedback and control overhead is reduced, the transmission efficiency is improved, and it can be adapted to different network environments and application requirements;
- A source location privacy protection scheme based on game theory is proposed. The interaction process between the attacker and the source node is described by evolutionary game equilibrium analysis, the balance point under different strategies is evaluated in time, and the defense strategy of the source node is dynamically adjusted to deal with multiple threats in time to ensure the security of source location privacy.

The rest of this paper is organized as follows: in Section 2, the related work of source location privacy protection is analyzed. In Section 3, the network model, underwater acoustic communication model and attack model used in this research are described. In Section 4, the design of the SLP-MACGT model is introduced in detail. In Section 5, the experimental simulation and performance analysis of SLP-MACGT are carried out. In Section 6, conclusions are drawn, and future research directions are discussed.

## 2. Related Work

In the rapidly evolving field of underwater acoustic sensor networks, ensuring the security and privacy of data transmission is of paramount importance. Researchers have been exploring innovative techniques and strategies to protect sensitive information and maintain the integrity of communication in challenging underwater environments. This section provides an overview of the existing work in source location privacy protection and sets the stage for the novel contributions presented in this paper.

### 2.1. Source Location Privacy Protection in WSNs

In the early 2000s, a series of seminal works contributed to advancing the concept of source location privacy in wireless sensor networks. In 2004, Ozturk et al. proposed the concept of source location privacy for the first time [16], mainly discussing the problem of protecting the source sensor location privacy in energy-constrained sensor networks, and proposed a flexible routing strategy named “phantom routing”. Building on this foundation, Koh et al. discussed the problem of optimal privacy protection probability routing in wireless networks. They proposed a novel privacy protection routing algorithm (OPERA) [17], aiming to optimize routing protocol privacy while considering utility constraints. Subsequently, they discussed the privacy utility trade-off issue and proposed a statistical decision-making framework to solve this problem. However, this method requires a lot of prior knowledge of location privacy routing, which is not easy to apply.

Continuing the exploration of privacy-preserving routing strategies, Chen et al. proposed a protection scheme based on Sector Phantom Routing (PSSPR) [18]. By utilizing the coordinates of the central node V to divide the sector area, phantom nodes execute the specific routing strategy to ensure that they can choose a variety of locations. Directed random routing ensures that data packets avoid visible areas when they move to the receiving node one by one and protects the source location privacy. Additionally, Sun et al. proposed a multi-path source location privacy protection scheme based on proxy nodes [19]. The sensor network is divided into four quadrants, and the source node initiates constrained flooding and directed routing algorithms to keep data packets away from the source location. By employing greedy quantitative routing, h-hop directional routing, and multi-path irregular spiral routing, the scheme makes it more difficult for attackers to backtrack paths, achieving a high level of privacy protection for the source location.

In a related development, Wu et al. proposed a game theory-based mobile location privacy protection [20], showing the necessity of game theory in solving the problem of mobile location privacy, proposed the classification of location privacy games among adversaries, data owners and collectors, and investigated and summarized the game models and equilibrium analysis in mobile location privacy. However, the authors treat service providers in mobile networks as untrustworthy, and each data owner needs to consider its own location privacy, which may lead to insufficient attention to the trust problem of service providers, and most of the privacy games are non-cooperative. Although significant progress has been made in wireless sensor networks, these source location privacy protection methods are not directly applicable to underwater network environments.

### 2.2. Source Location Privacy Protection in UASNs

In recent advancements in underwater sensor network research, several innovative source location privacy protection schemes have been proposed to address the unique challenges of securing data transmission in aquatic environments. Han et al. incorporated SLP into UASNs as the basis for a novel stratification-based source location privacy (SSLP) scheme [21]. SSLP is protected through the cooperation of autonomous underwater vehicles (AUVs) at each network layer. Similar to WSN, SSLP adds a false source node to the underwater cluster structure to increase the randomness of the underwater network. Additionally, false data streams are included in the AUV data collection and transmission of each cluster. However, the introduction of false source nodes and data streams can easily interfere with the transmission of normal data. Building on this concept, Wang et al. proposed a push-based probabilistic approach for source location privacy protection (PP-SLPP) [22]. PP-SLPP uses the pseudo-packet technique and multi-path technique to counteract passive attacks and uses the Ekman drift current model to divide the underwater environment into dynamic and static layers. Data collection of autonomous underwater vehicle (AUV) swarms is realized by hierarchical clustering and position pushing. However, the addition of pseudo-data packets and the strategy of AUV data collection will greatly increase the data transmission delay.

In parallel efforts, Liu et al. proposed a source location privacy protection scheme based on false packet time slot allocation (FPTSA-SLP) [23]. The time slot allocation mode is adopted to avoid interference with source data packets. Additionally, a handshake-based relay node selection method is also proposed to increase the diversity of routing paths to confuse adversaries. Based on reference [23], a conflict-free transmission-based source location privacy protection scheme (CFTSLP-TSA) was proposed in reference [24], selecting appropriate false source nodes to generate false data packets to conceal the traffic of source data packets. Subsequently, pseudo-sources and false data packets were arranged in different transmission time slots to prevent interference between each other. The handshake-based relay node selection strategy was used to diversify the paths, thereby imposing higher requirements on attackers for tracing the flow of source packets. However, the introduction of false sources and false data packets will increase the network’s load.

In a more sophisticated approach, Wang et al. proposed a layered source location privacy protection scheme based on network coding (SSLP-NC) [25]. To address two scenarios where the source is located in shallow sea and deep sea, distinct pseudo-source selection algorithms were proposed. Each node utilizes a pseudorandom number generator to periodically generate sequences to achieve the time-division transmission of real and false data. Considering the ability of malicious nodes to decode data packets, nodes employ existing pseudorandom number sequences as encoding vectors to encrypt both the source and false data to prevent adversaries’ decryption attempts. However, the capacity of linear network coding is limited, and currently, effective schemes for the integration of network coding with underwater source location privacy protection are still lacking.

Some achievements have been made in the research of underwater source location privacy protection schemes, but due to the complexity and uncertainty of underwater sensor networks, these schemes still need further research and improvement to enhance their practical application value. Currently, schemes designed for underwater source location privacy protection use the obfuscation technology of false sources and packets that increase communication and computation overheads, leading to network performance degradation. In addition, existing research focuses on resisting passive attacks, and there are a lack of strategies for resisting active attacks, which necessitate continuous updates and improvements. This paper comprehensively considers privacy protection, delay, and energy issues and presents a game theory-based underwater source location privacy protection scheme to address the aforementioned problems.

## 3. System Architecture and Assumptions

In this section, the network model, underwater acoustic communication model, and attack model used by the system are presented.

### 3.1. Network Model

**Figure 1:**
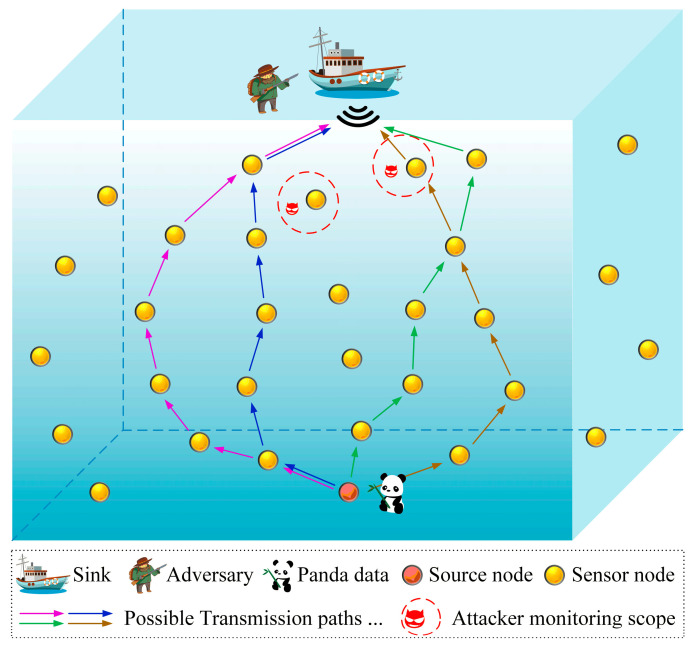
Network model.

In this paper, a spatial model of a 1000 m × 1000 m × 1000 m underwater acoustic network is constructed based on the classical panda hunter model [26], as illustrated in Figure 1. Sensor nodes are randomly placed in the underwater environment for sensing and transmitting data packets. Each sensor node is equipped with a depth sensor. Assuming that the network time is synchronized, all nodes work in half-duplex mode, transmitting and receiving packets at different time periods [27]. In addition, each node possesses a unique ID number and the same transmission capacity, buffer space, and computational power. The sink node is a strong and stable node deployed in the middle of the water, which is the ultimate destination of all source packets. An attacker lurks near the sink node but is unable to attack objects within a one-hop range from the sink node due to the region’s robust surveillance capability [28]. Once the target object is detected, the sensor node closest to the object is promptly activated to serve as the source node and reports the information about the monitored object to the sink node. It is assumed that the monitored objects are randomly displayed within the network and only one node acts as the source node at any given time. Any two sensor nodes in the network communicate in either one-hop or multi-hop mode. It is assumed that each node has N transmission frequencies, and each node can switch its frequency to send or receive packets.

### 3.2. Underwater Acoustic Communication Model

The communication methods commonly used on land, such as electromagnetic waves, infrared rays, and wireless signals, are not suitable for underwater environments due to propagation loss, multi-path interference, and spectrum limitations. Underwater acoustic propagation is suitable for underwater environments because it has characteristics such as long propagation distance and low loss, which allows underwater acoustic sensor networks to play an important role in underwater applications. The velocity of underwater acoustic propagation is affected by the properties of the medium such as temperature, pressure, salinity, and considering these factors comprehensively, the propagation velocity of an acoustic wave in an underwater environment can be expressed by the following equation [29]:

(1) $$\begin{array}{l} V=1449+4.591T-5.304\times 10^{-2}T^{2}+2.374\times 10^{-4}T^{3}\\ +1.34\left(S-35\right)+1.63\times 10^{-2}D+1.675\times 10^{-7}D^{2}\\ +1.025\times 10^{-2}T\left(S-35\right)-7.139\times 10^{-3}TD^{3} \end{array}$$

where T represents the temperature in degrees Celsius (°C), S represents the salinity in practical salinity units (PSU), and D represents the depth in meters (m).

### 3.3. Multiple-Attacker Model

It is assumed that the attacker lurks near the sink node. Once a new message is captured by the attacker, it will execute eavesdropping and traceback attacks [30]. Furthermore, this paper considers that the attacker carries out targeted active attacks in the case of passive attacks. The attacker has the capability of traffic analysis and packet deconstruction, including the ability to infer sensor node locations, and it can analyze and process eavesdropped data, deducing source locations by analyzing location information and communication data from multiple sensor nodes.

In previous research on the source location privacy protection of UASNs, scholars often consider that there is only a single attacker within the network. However, in actual situations, there may be multiple attackers who may collaborate with other attackers to jointly implement attack actions to achieve a common attack purpose. Forms of attacker cooperation include the division of labor cooperation, where each attacker is responsible for specific aspects or links of an attack; information sharing, where attackers enhance their attack abilities by sharing techniques, strategies, and information; and coordinated attacks, where multiple attackers act together with coordinated strategies and actions to carry out an attack. The differences between single-attacker and multiple-attacker scenarios are shown in Table 1.

It is worth noting that the attacker’s actions do not cause any functional interference to the network, such as adding routing paths, changing packets, damaging sensor nodes, and so on. Additionally, the attacker can only monitor the area within the receiving range of its device. This paper assumes that the attacker’s monitoring range is equal to the communication radius of the sensor node. Attackers use this eavesdropping radius to listen to the traffic in the network and try to detect the activity of the source node from it.

## 4. SLP-MACGT Model Design

The SLP-MACGT model’s mechanism involves the following steps: (1) network initialization, where each node obtains information about its hop count with the sink node and the neighbor table, including neighbors within two hops; (2) based on the virtual coordinate system, a node obtains the virtual position coordinates of itself and its neighbor nodes; (3) the relay nodes are selected based on the relay node selection strategy; (4) data packets are transmitted based on adaptive coding; and (5) the source node and the attacker complete the multi-round game until the source is changed or is found by the attacker.

### 4.1. Network Initialization

Nodes in underwater acoustic sensor networks need to be initialized, which mainly includes establishing a neighbor table and obtaining the hop count between themselves and the sink node. The sink node establishes a horizontal Cartesian coordinate system centered on itself and sends broadcast packets $\left\{ID,HopCount\right\}$ to all sensor nodes in Flooding mode, where ID indicates the sending node, and HopCount indicates the minimum hop count from the sink to this node. If the node receives a message from the sink for the first time, it records the hop count, updates the value of HopCount to HopCount+1, and sends the beacon message to its neighboring nodes. For each node that receives sink information, the ID and HopCount of the forwarding node should be stored in its neighbor table. When the Flooding is completed, each node records the sink and its own coordinate information, and neighboring nodes record and store the minimum value of HopCount from the sink.

### 4.2. Transformation Method Based on a Virtual Coordinate System

**Figure 2:**
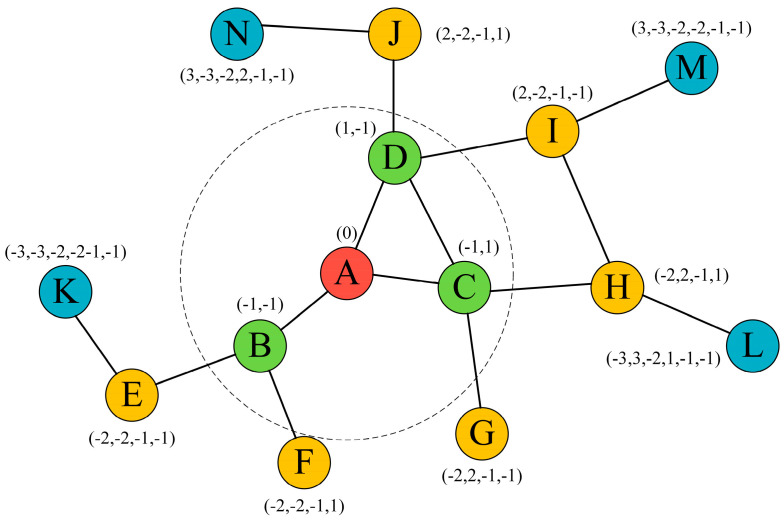
Setting virtual coordinates.

The virtual coordinate system is a technique used to locate nodes in a network. It does not rely on global location information, but it uses relative coordinates to represent the positional relationships between nodes. In this paper, the virtual coordinate system is combined with underwater acoustic sensor networks to protect the source location privacy, assuming that the location information of sensor nodes can be obtained by existing localization algorithms [31]. In underwater acoustic sensor networks, each node can generate virtual coordinates using its own physical location and the relative location information of neighboring nodes. Graph theory is used to model the communication relationships between nodes in underwater acoustic sensor networks in which sensor nodes can be represented as nodes in the graph and the relationships between sensors can be represented as edges in the graph, namely, $G=\left(V,E\right)$. As shown in Figure 2, the graph is traversed according to a breadth-first search to determine virtual coordinates for each node. First, node A is selected as the initial node and given the coordinate (0). All the neighbors of node A are found, and the neighbor nodes are numbered according to the binary representation. For example, node A has three neighboring nodes B, C, and D with numbers 00, 01, and 10, respectively, and the coordinates are (−1, 1), (−1, 1), and (1, 1), respectively. Node B has two neighbors, E and F, whose numbers are 00 and 01 and whose coordinates are (−2, −2, −1, −1) and (−2, −2, −1, 1), respectively. Node C has three neighbors D, G, and H. Since node D has virtual coordinates, the coordinates of node D are unchanged, and the coordinates of neighboring nodes G and H are (−2, 2, −1, −1), and (−2, 2, −1, 1), respectively. The remaining nodes are traversed by the same graph traversal method to determine their virtual coordinates.

The nodes maintain a mapping table that corresponds virtual coordinates to actual locations. These virtual coordinates are only used for internal calculations and do not reveal actual geographic location. When data need to be transmitted, a node can use the virtual coordinate system to select the next-hop transmission node. During data transmission, nodes can map virtual coordinates back to actual geographic coordinates to determine the actual transmission path of the data. This mapping process should take place inside the node and does not need to be propagated outside. Before the source node sends data, the virtual coordinates of the source node can be obfuscated or encrypted to protect the source location’s privacy. Only the nodes inside the network know how to decrypt or restore these coordinates.

Combined with the virtual coordinate system, the underwater acoustic sensor network can achieve source location privacy protection to a certain degree. The virtual coordinate system allows nodes in the network to perform routing and data transmission according to their relative positions without revealing actual geographic coordinate information, ensuring that the geographic locations of the source nodes are not easily determined by external malicious parties or potential attackers. Moreover, each node only knows the location of itself and its neighboring nodes and does not need to obtain the location information of all nodes in the network, which saves storage space and improves security.

### 4.3. Relay Node Selection Strategy

In order to further protect the location privacy of the source node and make the actual location of the source node more difficult to observe or trace externally, the selection of relay nodes is very important. Once panda data are monitored, the source node periodically generates packets and transmits them to the sink node via multiple hops. The strategy proposed in this paper is divided into four distinct stages and is shown in Algorithm 1.

1. Limited Flooding: The source node uses limited Flooding to transmit messages within the monitoring range, and the number of hops is limited to H to achieve directional routing. Once the target enters the monitoring range, the source node sets a timer and broadcasts the message $Smessage=\left\{ID,Stime\right\}$ to the nodes within the range of H hops, and the ID of the sensor node is used as a unique identifier. Stime is set to the maximum transmission time H, which decreases with the transmission time until it reaches zero, when the receiving node stops forwarding messages. A node ID_v receiving message Smessage is marked as visible if its $Hop_{count}$ is less than R (communication radius < eavesdropping distance). During the Flooding process, each node receiving message Smessage can obtain the minimum hop count from the source node to the node itself;
2. H-hop-directed routing: According to the minimum number of hops from neighboring nodes to the source node, the next-hop node is selected for H-hop-directed routing to participate in packet forwarding. The forwarding time starts at zero and is increased by one for each execution until H is reached. The farthest H-hop neighbor node acts as participant M and is responsible for forwarding the packet from the source node;
3. Greedy quantitative routing: The length of greedy quantitative routing is defined as L, and the relay node N forwards the data packet to the sink node with a transmission time of L. In this process, care should be taken to randomly select a relay node N from the unseen region;
4. Multi-path forwarding routing based on relay nodes: Node N generates angle τ, where $\tau \in \left[0,\pi \right]$, and randomly completes a variable length equal-hop path $H_{m}$ in the counterclockwise or clockwise direction to reach the next relay node O. Node O randomly selects an *I*-step equal-hop route to reach the sink node.

**Algorithm 1:** Relay Node Selection Strategy Input: Source Node, Communication Radius, Monitoring Range Output: Selected Relay Nodes Limited Flooding: 1: Set the maximum transmission time Stime to a predefined value; 2: Initialize the hop count H to limit the number of hops for directional routing; 3: Broadcast a message within the monitoring range; 4: Nodes receiving the message mark themselves as visible if their distance is less than the communication radius R; 5: Calculate the minimum hop count from the source node to each visible node; Relay Node Selection: 6: Identify relay nodes based on the minimum hop count and visibility; 7: The farthest neighbor node of the H hop acts as participant M and forwards the data packet away from the source node; 8: The node that forwards the packet to the Sink node by forwarding time L is selected as the next relay node N; 9: The relay node N generates angle τ and randomly completes a variable length equal-hop path to reach the next relay node O; 10: Node O randomly selects an *I*-step equal-hop route to reach the sink node; 11: Ensure the relay node is positioned to obscure the actual location of the source node; Path Establishment: 12: Establish secure transmission paths through selected relay nodes; 13: Ensure data packets are forwarded through relay nodes to reach the sink node; 14: Maintain the integrity and confidentiality of data transmission; End of Transmission: 15: Stop the transmission process once data packets reach the sink node.

The selection of relay nodes has an important impact on the safety time. In network communication, safety time is the time required for data to travel from the source node to the destination node, including the data transmission and forwarding time of the relay node. Selecting appropriate relay nodes can optimize the data transmission path, prolong the safety time, and improve the efficiency and reliability of data transmission.

Utilizing H-hop-directed routing forwards packets away from the source. Greedy quantitative routing is used to expand the optional range relay node N. It can be seen that there are $4\pi H^{2}$ forwarding paths generated in these phases. During the relay node-based multi-path forwarding routing phase, the relay node O may be positioned in any direction relative to the sink node. It can be seen that there are $4\pi l^{2}$, where $l\in \left[Hop_{source-\sin k}-H-L,Hop_{source-\sin k}+H-L\right]$, forwarding paths generated in these phases. Therefore, the number of paths generated by the relay node selection strategy is $4\pi H^{2}\times 4\pi l^{2}=16\pi^{2}H^{2}l^{2}$ and the probability of path duplication is $\frac{1}{16\pi^{2}H^{2}l^{2}}$.

### 4.4. Secure Data Transmission Based on Fountain Codes

In the previous section, we realized the multi-path transmission of data packets through the selection of relay nodes mainly to resist eavesdropping attacks and backtracking attacks. The primary objective of this section is to resist an active attack during a passive attack. For a powerful attacker who can record eavesdropping packets and mine them for analysis, encrypted packets are insufficient to protect source location and monitor target location privacy. Therefore, the algorithm of this paper combines network coding technology and proposes a secure data transmission method based on fountain codes to resist active attacks. Luby transform codes (LT codes) are used to encode the data packets sent by the source, which is an error-correcting coding method designed to achieve high error tolerance [32]. Unlike traditional error-correcting codes, fountain codes are characterized by the ability to generate an unlimited number of coded symbols, which makes them superior in unreliable underwater acoustic communication.

#### 4.4.1. Data Encoding

In the data transmission phase, the message to be transmitted by the source node is first divided into $K\left(s_{1},s_{2},\ldots ,s_{K}\right)$ packets, and the length of each packet is n. The degree $d_{n}$ of each code element is randomly selected by a random number generator according to a specific degree probability distribution function $\rho \left(d\right)$, and $1\leq d_{n}\leq K$ represents the number of packets that a set of messages must contain. The $d_{n}$ code elements involved in encoding are also random, and the output of the encoder $t_{n}$ is obtained from an XOR operation of any $d_{n}$$s_{k}$.

The degree distribution function of LT codes is determined by the ideal solitary wave distribution first proposed by Luby [32]:

(2) $$\rho \left(d\right)=\left\{\begin{array}{cc} \frac{1}{K}, & d=1\\ \frac{1}{d\left(d-1\right)}, & d=2,3,\ldots ,K \end{array}\right.$$

**Figure 3:**
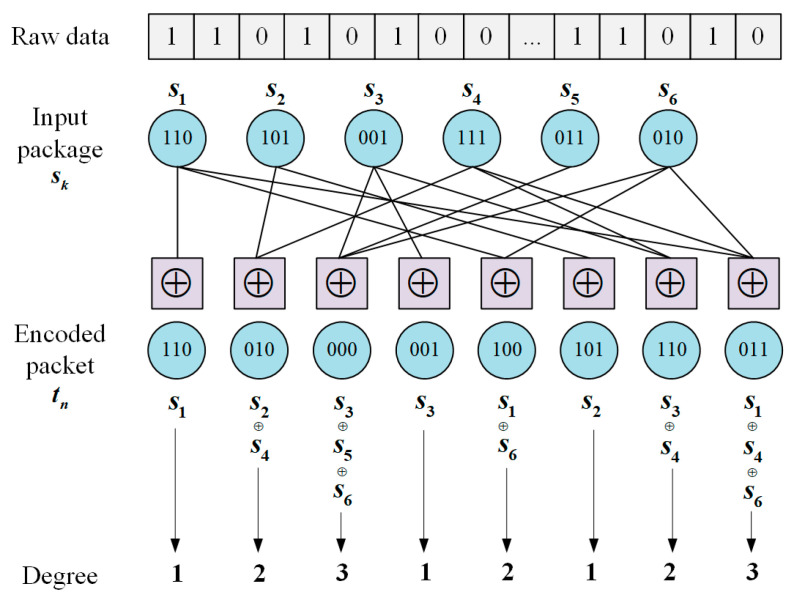
Data encoding process.

After determining the degree of an encoding, it is also necessary to determine which $s_{k}$ values participate in the XOR coding operations. $s_{k}$ can be selected in the same way as the determination of degree. The encoding process of LT codes is shown in Figure 3.

#### 4.4.2. Data Decoding

**Figure 4:**
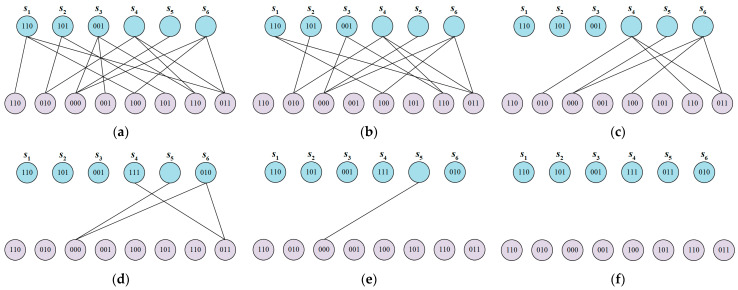
Data decoding process: (**a**) the received coded packets; (**b**) recovering *S*_1_, *S*_2_, *S*_3_; (**c**) removing the edges connected to *S*_1_, *S*_2_, *S*_3_; (**d**) recovering *S*_4_, *S*_6_; (**e**) removing the edges connected to *S*_4_, *S*_6_; (**f**) recovering *S*_4_, *S*_6_.

After receiving an encoded packet, the receiver finds a check code $t_{n}$ with a degree of 1. If there is no such check code, decoding terminates, and the source files cannot be recovered. Next, let $s_{k}=t_{n}$, carry out XOR operations between $s_{k}$ and all $t_{n}$ that are connected to $s_{k}$, and delete all connections with $s_{k}$. The above procedure is repeated until all $s_{k}$ are determined. The decoding process of LT code is shown in Figure 4. First, an encoded packet with a degree of 1 is found, which is used as a check code; $s_{1},s_{2},s_{3}$ with degrees of 1 are found as shown in the figure; and $s_{1},s_{2},s_{3}$ are determined to be 110, 101, and 001, respectively. Since $s_{1},s_{2},s_{3}$ are also related to $s_{4},s_{6}$, XOR is performed between $s_{1},s_{2}$ and $s_{4},s_{6}$, respectively; hence, $s_{4},s_{6}$ are 111 and 010, and all links with $s_{1},s_{2},s_{3}$ are disconnected at the same time. Then, the search for a check code of degree 1 is continued, $s_{6}$ is found, and the above decoding process is repeated until all $s_{k}$ values are determined.

The reliability of data transmission can be improved by the introduction of redundant data, the security of data transmission can be increased through using the random coding method, and the risk of information leakage and tampering during data transmission is reduced by the forward error correction feature of the LT code. The secure data transmission method based on fountain codes adapts to a complex transmission environment, provides effective support for the secure transmission of underwater acoustic sensor networks and effectively resists active attacks during passive attacks.

### 4.5. Game Process between Sensor Nodes and Attackers

Source location privacy protection is an important issue in underwater acoustic communication and location-based services. The source node aims to protect its location information, while the attacker tries to obtain this information, and this competitive relationship can be modeled and analyzed by game theory. Game theory can play an important role in quantifying source location privacy and safety time and can be used to model and analyze the interactions between source nodes and potential attackers to determine the best privacy protection strategy. Through the analysis of game theory, the balance point of the strategy can be found to achieve the best trade-off between privacy protection and time efficiency, which helps to ensure that source location privacy is fully protected within a given safe time.

#### 4.5.1. Basic Assumptions of the Game Model

The relationship between the source node and the attacker can be regarded as a zero-sum game. In general, the source node knows its own position, while the attacker can only estimate or infer the position information through certain methods [33]. In this paper, we consider a scenario where there are multiple attackers in the network, involving interactions and decisions among multiple participants. Source location privacy in underwater acoustic sensor networks usually involves multiple rounds of the game, which includes repeated strategy selection, confrontation, and adjustment between the source node and attackers. The strategies of source nodes and attackers are often dynamic and can evolve over time, and evolutionary game theory can capture these changes [34] and allow analysis strategies to adapt to changing environments over time. Unlike traditional static games, evolutionary games focus on how players choose strategies in multiple rounds of the game, and these strategies can spread and mutate through a series of evolutionary mechanisms. At the same time, evolutionary game theory no longer treats both players of the game as superrational players but believes that players usually reach game equilibrium through trial and error.

The main components of game theory include several elements: players, game strategies, and game benefits.

The participant set is $PLAYER=\left(player_{X},player_{Y},player_{Z},\ldots \right)$. The source node $\left(player_{X}\right)$ is a player that actively protects its location privacy in the network, and its goal is to select a strategy that maximizes location privacy while maintaining communication performance. This paper takes the existence of two attackers as an example: A $\left(player_{Y}\right)$ and B $\left(player_{Z}\right)$ are two players who actively try to obtain the location information of the source node, and the goal is to choose a strategy to infer the location of the source node to the greatest extent.

The game strategy set is $STR=\left(S_{X},S_{Y},S_{Z}\right)$. $S_{X}$ is the strategy space of the source node, and the source node can choose different protection strategies, such as a virtual coordinate system, relay node selection, and network coding, to form the source node strategy space $S_{X}=\left\{X_{1},X_{2}\right\}$ (weak defense, strong defense). $S_{Y}$ is the strategy space of attacker A, who can adopt methods, such as eavesdropping attack, traceback attack, traffic analysis, or cooperation with other attackers, to form the attacker’s strategy space $S_{Y}=\left\{Y_{1},Y_{2}\right\}$ (single attack, cooperative attack). The strategy space of attacker B, $S_{Z}=\left\{Z_{1},Z_{2}\right\}$ (single attack, cooperative attack), is the same as that of attacker A. Only when both attackers choose a cooperative strategy can cooperation between the attackers be achieved.

The probability $P=\left(P_{X},P_{Y},P_{Z}\right)$ of game strategy selection is the probability set of the source node and attacker strategies, where $P_{X}=\left\{x,1-x\right\}$ represents the probability of weak defense and strong defense of the source node, $P_{Y}=\left\{y,1-y\right\}$ represents the probability of attacker A’s single attack and cooperative attack, and $P_{Z}=\left\{z,1-z\right\}$ represents the probability of attacker B’s single attack and cooperative attack.

The game return can be set as $RETURN=\left(R_{X},R_{Y},R_{Z}\right)$. $R_{X}$ represents the return set of the source node, $R_{Y}$ represents the return set of attacker A, and $R_{Z}$ represents the return set of attacker B. The return of the source node is measured by the information entropy, and the return of the attacker is measured by the information gain, indicating that the attacker’s uncertainty about the location of the source node is reduced after obtaining a certain amount of information.

The game cost can be set as $COST=\left(C_{X},C_{Y},C_{Z}\right)$. $C_{X}$ represents the payoff set of the source node, $C_{Y}$ represents the payoff set of attacker A, and $C_{Z}$ represents the payoff set of attacker B. The energy consumption of a node represents the game cost for the source node and the attacker.

#### 4.5.2. Establishment of an Evolutionary Game Model

According to the behavioral strategies of the source node and the attacker, it can be concluded that there are eight kinds of game combinations. In game theory, the payoff usually refers to the utility or benefit that the parties involved in the game can obtain under different strategies. The game gain matrix for UASNs is derived based on the aforementioned definition in combination with the underwater acoustic sensor network model, as illustrated in Table 2.

#### 4.5.3. Replication Dynamic Equation of Tripartite Evolutionary Game

A key concept in game theory is equilibrium analysis. By studying the Nash equilibrium point of the game, insight can be gained into the interactions between the source node and the attacker and possible results. At the equilibrium point, no party can achieve better results by changing its strategy, which helps balance the trade-off between privacy protection and information access. Therefore, the payoff of the source node choosing a different strategy is

(3) $$\left\{\begin{array}{c} \begin{array}{l} G\left(X_{1}\right)=yz\left(R_{X1}-R_{Y1}-R_{Z1}-C_{X1}\right)+\left(1-y\right)z\left(R_{X1}-R_{Y2}-R_{Z1}-C_{X1}\right)\\ +y\left(1-z\right)\left(R_{X1}-R_{Y1}-R_{Z2}-C_{X1}\right)+\left(1-y\right)\left(1-z\right)\left(R_{X1}-R_{Y2}-R_{Z2}-C_{X1}\right) \end{array}\\ \begin{array}{l} G\left(X_{2}\right)=yz\left(R_{X2}-R_{Y1}-R_{Z1}-C_{X2}\right)+\left(1-y\right)z\left(R_{X2}-R_{Y2}-R_{Z1}-C_{X2}\right)\\ +y\left(1-z\right)\left(R_{X2}-R_{Y1}-R_{Z2}-C_{X2}\right)+\left(1-y\right)\left(1-z\right)\left(R_{X2}-R_{Y2}-R_{Z2}-C_{X2}\right) \end{array} \end{array}\right.,$$

The average revenue of the source node is as follows: $\bar{G\left(X\right)}=xG\left(X_{1}\right)+\left(1-x\right)G\left(X_{2}\right)=\sum_{i}P_{x}G\left(X_{i}\right)$. The replication dynamic equation of the source node is

(4) $$F\left(x\right)=\frac{dx\left(t\right)}{dt}=x\left(G\left(X_{i}\right)-\bar{G\left(X\right)}\right).$$

The payoff for attacker A in choosing a different strategy is

(5) $$\left\{\begin{array}{c} \begin{array}{l} G\left(Y_{1}\right)=xz\left(R_{Y1}-R_{X1}-C_{Y1}\right)+x\left(1-z\right)\left(R_{Y1}-R_{X1}-C_{Y1}\right)\\ +\left(1-x\right)z\left(R_{Y1}-R_{X2}-C_{Y1}\right)+\left(1-x\right)\left(1-z\right)\left(R_{Y1}-R_{X2}-C_{Y1}\right) \end{array}\\ \begin{array}{l} G\left(Y_{2}\right)=xz\left(R_{Y2}-R_{X1}-C_{Y1}\right)+x\left(1-z\right)\left(R_{Y2}-R_{X1}-C_{Y1}\right)\\ +\left(1-x\right)z\left(R_{Y2}-R_{X2}-C_{Y1}\right)+\left(1-x\right)\left(1-z\right)\left(R_{Y2}-R_{X2}-C_{Y1}\right) \end{array} \end{array}\right.,$$

The average revenue of attacker A is as follows: $\bar{G\left(Y\right)}=yG\left(Y_{1}\right)+\left(1-y\right)G\left(Y_{2}\right)=\sum_{i}P_{y}G\left(Y_{i}\right)$. The replication dynamic equation of attacker A is as follows:

(6) $$F\left(y\right)=\frac{dy\left(t\right)}{dt}=y\left(G\left(Y_{i}\right)-\bar{G\left(Y\right)}\right).$$

The payoff for attacker B in choosing a different strategy is

(7) $$\left\{\begin{array}{c} \begin{array}{l} G\left(Z_{1}\right)=xy\left(R_{Z1}-R_{X1}-C_{Z1}\right)+x\left(1-y\right)\left(R_{Z1}-R_{X1}-C_{Z1}\right)\\ +\left(1-x\right)y\left(R_{Z1}-R_{X2}-C_{Z1}\right)+\left(1-x\right)\left(1-y\right)\left(R_{Z1}-R_{X2}-C_{Z1}\right) \end{array}\\ \begin{array}{l} G\left(Z_{2}\right)=xy\left(R_{Z2}-R_{X1}-C_{Z2}\right)+x\left(1-y\right)\left(R_{Z2}-R_{X1}-C_{Z2}\right)\\ +\left(1-x\right)y\left(R_{Z2}-R_{X2}-C_{Z2}\right)+\left(1-x\right)\left(1-y\right)\left(R_{Z2}-R_{X2}-C_{Z2}\right) \end{array} \end{array}\right.,$$

The average revenue of attacker B is as follows: $\bar{G\left(Z\right)}=zG\left(Z_{1}\right)+\left(1-z\right)G\left(Z_{2}\right)=\sum_{i}P_{z}G\left(Z_{i}\right)$. The replication dynamic equation of attacker B is as follows:

(8) $$F\left(z\right)=\frac{dz\left(t\right)}{dt}=z\left(G\left(Z_{i}\right)-\bar{G\left(Z\right)}\right).$$

Combining the above Equations (4), (6) and (8) into Equation (9), the replication dynamic equation system is established as follows:

(9) $$Y=\left[\begin{array}{c} F\left(x\right)\\ F\left(y\right)\\ F\left(z\right) \end{array}\right]=f\left(Y,t\right)=0.$$

Solving the equation yields: $Y_{1}=\left[\begin{array}{c} 0\\ 0\\ 0 \end{array}\right]$, $Y_{2}=\left[\begin{array}{c} 1\\ 0\\ 0 \end{array}\right]$, $Y_{3}=\left[\begin{array}{c} 0\\ 1\\ 0 \end{array}\right]$, $Y_{4}=\left[\begin{array}{c} 0\\ 0\\ 1 \end{array}\right]$, $Y_{5}=\left[\begin{array}{c} 1\\ 1\\ 0 \end{array}\right]$, $Y_{6}=\left[\begin{array}{c} 1\\ 0\\ 1 \end{array}\right]$, $Y_{7}=\left[\begin{array}{c} 0\\ 1\\ 1 \end{array}\right]$, and $Y_{8}=\left[\begin{array}{c} 1\\ 1\\ 1 \end{array}\right]$.

#### 4.5.4. Nash Equilibrium of the Tripartite Game

Each equilibrium point in the system corresponds to an evolutionary game equilibrium. Table 3 shows the eigenvalues corresponding to all equilibrium points.

Using the indirect Lyapunov method, if all the eigenvalues of the Jacobian matrix have a negative real part, the equilibrium point is an asymptotically stable point. When $C_{X2}-C_{X1}+R_{X1}-R_{X2}>0$, $C_{Y2}-C_{Y1}+R_{Y1}-R_{Y2}>0$, and $C_{Z2}-C_{Z1}+R_{Z1}-R_{Z2}>0$ are satisfied, the above eight equilibrium points are all stable.

## 5. Experimental Simulation and Analysis

### 5.1. Simulation Setup

In this paper, the performance of the SLP-MACGT model is compared with that of the stratification-based source location privacy scheme (SSLP) [11], the push-based probabilistic method source location privacy scheme (PP-SLPP) [12], and the multi-round game-based source location privacy scheme (MRGSLP) [35]. Simulations were performed using MATLAB 2021 to evaluate the performance of these models. In the simulations, all sensor nodes are randomly distributed in a space of 1000 m × 1000 m × 1000 m with the default simulation parameters listed in Table 4.

The performance evaluation indexes used in this paper are

- Packet delivery ratio (PDR): PDR is the probability of successfully forwarding data packets from the source node to the sink node;
- Network safety time: Network safety time refers to the time between the activation of the source node and the successful detection of the source node by the attacker under the premise that the source node continues to send packets;
- End-to-end delay: End-to-end delay represents the time required to transmit a data packet from a source node to the sink node;
- Energy consumption: Energy consumption represents the energy consumption of transmitting and receiving data and controlling data packets during a simulation run.

According to Xing et al. [36], the energy consumed by a sensor node to send a data packet with l bits can be expressed as follows:

(10) $$E_{sent}\left(l,d\right)=lP_{0}A\left(d,f\right)=lP_{0}d^{k}\alpha {\left(f\right)}^{d},$$

where $P_{0}$ is the received power level of the node, $A\left(d,f\right)$ is the power attenuation coefficient relevant to the distance, k is the spreading factor of the propagation geometry, and $\alpha \left(f\right)$ is the acoustic signal absorption coefficient.

The energy consumed by the node to receive l bits data can be calculated by

(11) $$E_{received}\left(l\right)=lE_{1}.$$

where $E_{1}$ represents the reception coefficient, which is the energy consumption for receiving 1 bit data.

### 5.2. Performance of the SLP-MACGT Model

#### 5.2.1. Effect of the Network Side Length on Performance

**Figure 5:**
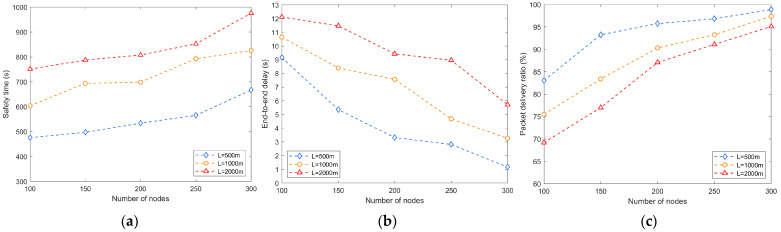
Effect of network side length on performance: (**a**) safety time; (**b**) end-to-end delay; (**c**) packet delivery ratio.

The network scale plays an important role in network performance. When the network scale increases, such as increasing the number of nodes or the side length of the network, it will directly affect the capacity, coverage, transmission efficiency and data security of the network. L represents the network side length, which is the length, width and height of the network space. In the following, we explore the impact of different network sizes (L = 500 m, 1000 m, and 2000 m) on source location privacy protection. As shown in Figure 5a, increasing the number of nodes can enhance the capacity and coverage of the network while improving the privacy protection of the source location when the length of the network side is fixed. As the scale of the network increases, the communication paths between nodes become longer, resulting in prolonged information transmission times. This may increase the time needed for attackers to obtain the source location information. Therefore, increasing the number of nodes and the side length of the network will result in a longer safety time.

As shown in Figure 5b,c, more nodes can provide more path choices, reduce congestion and network delay, and improve the transmission efficiency of data packets in the network, thus increasing the packet forwarding rate. However, the increase in network side length leads to longer transmission paths between nodes, which increases the delay of data transmission, negatively affecting real-time applications and delay-sensitive tasks. At the same time, a longer network side length increases the risk of instability and packet loss during data transmission. The packets need to pass through more nodes during forwarding and may face problems such as signal attenuation, interference, or node failure, which leads to a decrease in the packet delivery ratio.

#### 5.2.2. Effect of the Communication Radius on Performance

**Figure 6:**
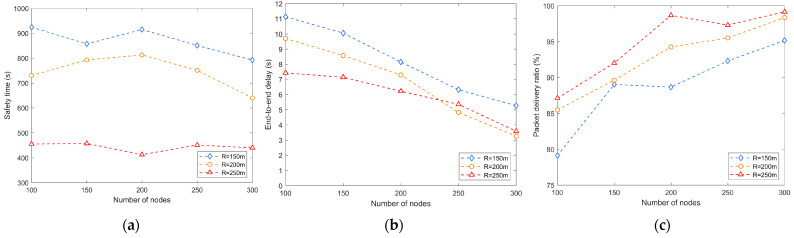
Effect of the communication radius on performance: (**a**) safety time; (**b**) end-to-end delay; (**c**) packet delivery ratio.

The following experiment investigates the effect of the communication radius on performance. As shown in Figure 6a, when the communication radius increases, the communication range between nodes expands, and the probability of an attacker contacting the network also increases. Therefore, a larger transmission radius may lead to a shorter safety time, and it will be easier for the attacker to obtain the source location information.

As shown in Figure 6b, a larger communication radius means that data may be delivered through fewer intermediate nodes, thus reducing latency. As shown in Figure 6c, the change in the communication range may affect the direct communication ability between nodes. If the communication range is reduced, the direct connections between nodes may decrease, resulting in a lower success rate of data forwarding. In contrast, an increase in the communication range may increase the direct connection between nodes and may help improve the data forwarding success rate.

#### 5.2.3. Effect of the Number of Attackers on the Safety Time

**Figure 7:**
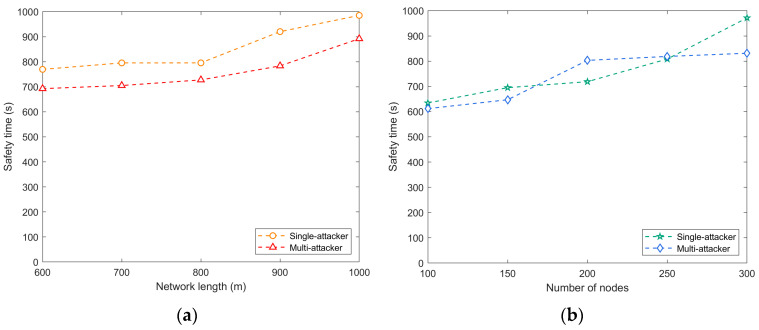
Safety time for a single attacker and multiple attackers: (**a**) network side length vs. safety time; (**b**) number of nodes vs. safety time.

As shown in Figure 7, it typically takes more time for a single attacker to successfully breach or compromise network security. They may need to conduct eavesdropping, analysis, and attack attempts for longer periods of time to identify weaknesses and obtain valuable data. In contrast, multiple attackers can attack different targets at the same time, taking advantage of cooperation to speed up the attack and shorten the time window for a successful attack. A larger network scale complicates the transmission path, and the attacker needs more time to obtain the source location information. In addition, a longer network side length may result in a longer signal transmission path, which increases the time required for the attacker to determine the source location.

### 5.3. SLP-MACGT Comparison with Other SLP Schemes

#### 5.3.1. Network Safety Time

**Figure 8:**
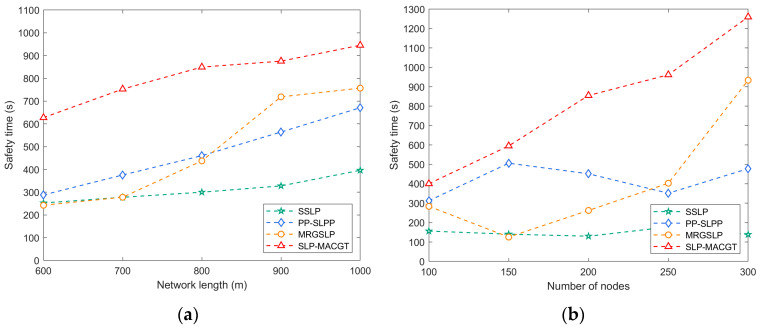
Safety time: (**a**) network side length vs. safety time; (**b**) number of nodes vs. safety time.

In the evaluation of network safety time depicted in Figure 8a, the influence of network side length on algorithm performance is striking. With the integration of multiple source location privacy protection strategies and the attainment of Nash equilibrium via evolutionary games, SLP-MACGT emerges as the frontrunner among the algorithms considered. Conversely, the safety times of SSLP and PP-SLPP exhibit dependence on AUV movement. When the network scale increases, the safety times of these three algorithms will suddenly increase.

Moreover, Figure 8b offers results into safety time dynamics concerning varying node counts. Despite fluctuations in the curves, a consistent trend emerges: both the SLP-MACGT algorithm and its counterparts exhibit an incremental rise in safety times with increasing sensor nodes. This trend stems from the heightened node density, which augments the number of available transmission paths, thereby enhancing path diversity and bolstering the overall network safety time. Notably, the performance of MRGSLP initially falters, but it gradually improves, which could be chiefly attributed to communication range adjustments.

#### 5.3.2. End-to-End Delay

**Figure 9:**
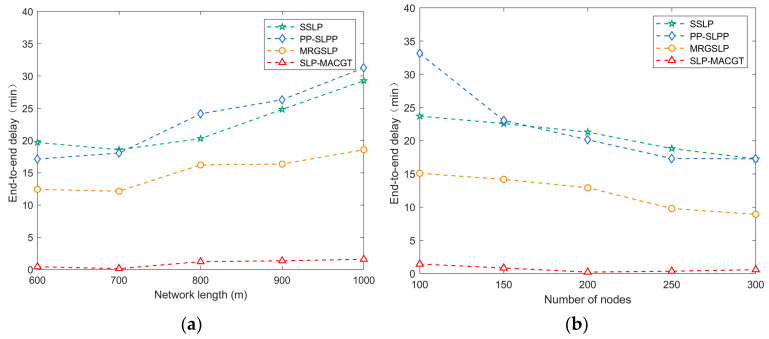
End-to-end delay: (**a**) network side length vs. end-to-end delay; (**b**) number of nodes vs. end-to-end delay.

Figure 9a shows that in terms of latency, SLP-MACGT exhibits significantly lower end-to-end latency compared to SSLP, PP-SLPP, and MRGSLP. While PP-SLPP, SSLP, and MRGSLP experience a notable increase in latency due to the utilization of AUVs, with latency on the order of minutes, the latency magnitude of SLP-MACGT is on the scale of seconds. Specifically, the higher latency in PP-SLPP compared to SSLP is attributed to the time taken for the leading AUV to collect data from all following AUVs, whereas in SSLP, AUVs collect data based on trajectories without significant delays. MRGSLP divides the network into static and dynamic layers, with nodes in the static layer requiring AUV assistance for data transmission.

Moreover, Figure 9a indicates that latency increases with the growth of the network edge length. In Figure 9b, the fluctuations in PP-SLPP latency stem from variances in pushing positions and random initial positions of AUV groups. The latency of SSLP increases with the rising number of nodes as AUVs collect data from more nodes along fixed trajectories. In SLP-MACGT, as the number of nodes increases, senders can choose the next hop nodes closer to the convergence node, resulting in a gradual decrease in latency as node count rises.

#### 5.3.3. Packet Delivery Ratio

**Figure 10:**
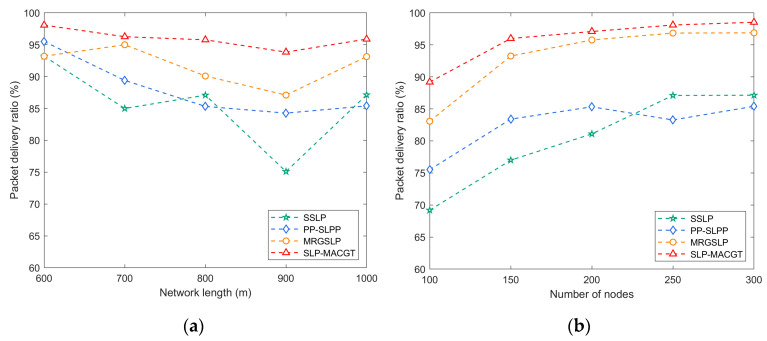
Packet delivery ratio: (**a**) network side length vs. packet delivery ratio; (**b**) number of nodes vs. packet delivery ratio.

Figure 10 shows that SLP-MACGT demonstrates the highest data packet transmission rate among the compared protocols. SLP-MACGT implements a secure data transmission strategy based on fountain codes, which enhances the success rate of data transmission while reducing the need for data retransmission. In PP-SLPP, AUVs primarily handle most of the network functionalities, and the data packet transmission rate in PP-SLPP is influenced by the collaboration between leading AUVs and their followers, as the leading AUVs gather data from all the following AUVs.

SSLP utilizes AUVs to collect data and necessitates periodic data relaying, potentially leading to higher node energy consumption, thereby influencing data packet transmission rates. As the number of nodes increases, AUVs collect data from more nodes along fixed trajectories, impacting data packet transmission rates. MRGSLP partitions the network into static and dynamic layers, with nodes in the static layer requiring assistance from AUVs for data transmission. This layering scheme may influence data packet transmission rates.

#### 5.3.4. Energy Consumption

**Figure 11:**
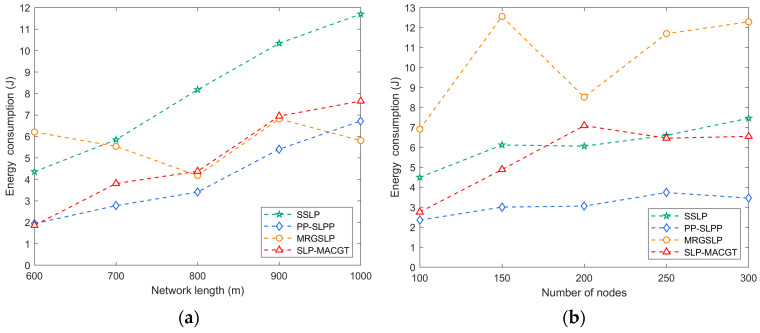
Energy consumption: (**a**) network side length vs. energy consumption; (**b**) number of nodes vs. energy consumption.

Figure 11a illustrates the comparison of node energy consumption among different methods, showcasing that the energy consumption of SLP-MACGT remains at an intermediate level even when facing multiple attackers. The incorporation of network coding and multi-round games in SLP-MACGT does impose a slight burden on the nodes; however, the variance in energy consumption between SLP-MACGT and MRGSLP is not notably significant. In contrast, the utilization of SSLP results in the highest energy consumption by nodes due to the traversal of multiple AUVs across all clusters and the energy requirements for periodic data relays. Conversely, PP-SLPP demonstrates relatively low energy consumption as several AUVs manage the majority of network functions, thereby consuming the bulk of the energy resources.

Furthermore, Figure 11b provides additional insights into the comparison of energy consumption across varying numbers of nodes. In SSLP and PP-SLPP, changes in the node count have minimal effects on energy consumption, as AUVs predominantly handle data transmission tasks. Conversely, in SLP-MACGT and MRGSLP, nodes are responsible for a significant portion of the network functionality, leading to heightened energy consumption during simulation scenarios. Notably, the adaptive coding strategy employed in SLP-MACGT enhances data transmission success rates, reduces the need for data retransmissions, and ultimately lowers overall energy consumption compared to MRGSLP.

## 6. Conclusions

Source location privacy protection is a challenging task in underwater acoustic sensor networks. This paper discusses the key challenges of source location privacy protection, designs for the scenario of multiple cooperative attackers, and proposes an underwater source location privacy protection scheme based on game theory. The scheme comprehensively considers privacy protection, delay and energy issues, and it effectively protects the source location privacy by means of virtual coordinate system transformation, relay node selection strategy and fountain code secure data transmission technology. The introduction of game theory as a framework allows strategic interaction between source nodes and malicious nodes, provides a new perspective for source location privacy protection in underwater acoustic sensor networks, and improves network security. The simulation results show that the proposed scheme achieves significant improvements in terms of packet delivery ratio, security time, delay and energy consumption. The packet delivery rate average increases by 30%, security time is extended by at least 85%, and the delay is reduced by at least 90% compared with SSLP, PP-LSPP, and MRGSLP, which provides strong support for the security and performance of underwater acoustic sensor networks.

However, there are some limitations to the proposed scheme. Due to the lack of real marine data, mathematical methods are currently the only means to quantify the benefits and costs of the game between source nodes and attackers. In the future, research needs to delve deeper into the cooperative patterns among multiple attackers to devise more effective response strategies. Considering simulation experiments and actual observational data for validating and optimizing the accuracy and practicality of the model, and further understanding the advantages and limitations of various methods, will be crucial in exploring more effective and reliable source location privacy protection mechanisms to ensure location privacy and data security in underwater acoustic sensor networks.

## Figures and Tables

**Table 1:** Comparison of single-attacker and multi-attacker capabilities.

Feature	Single Attacker	Multiple Attackers
Number of attackers	One attacker	Multiple attackers
Attack complexity	Usually independent operations, relatively simple attacks	More sophisticated coordinated attack strategies
Difficulty of detection	Easier to detect because it is a single entity	Complex to detect due to distributed actions
Network influence	Some privacy impact	Significant privacy threat, especially when acting in concert
Countermeasures to the attack	Easier to develop coping strategies	More sophisticated coping strategies may be required
Attack surface	Limited to the capabilities of a single attacker	Broader attack surface with diverse strategies and resources
Crypticity	Relatively easy to remain hidden and hard to detect	Difficult to remain completely hidden

**Table 2:** Game gain matrix.

Strategy combination	$\left(X_{i},Y_{j},Z_{k}\right)$
Payoff of source node gain	$R_{Xi}-R_{Yj}-R_{Zk}-C_{Xi}$
Payoff of attacker A gain	$R_{Yj}-R_{Xi}-C_{Yj}$
Payoff of attacker B gain	$R_{Zk}-R_{Xi}-C_{Zk}$

Where i,j,k=1 or 2.

**Table 3:** Eigenvalues at different equilibrium points.

The Equilibrium Point	Eigenvalue 1	Eigenvalue 2	Eigenvalue 3
$\left(0,0,0\right)$	$C_{X2}-C_{X1}+R_{X1}-R_{X2}$	$C_{Y2}-C_{Y1}+R_{Y1}-R_{Y2}$	$C_{Z2}-C_{Z1}+R_{Z1}-R_{Z2}$
$\left(1,0,0\right)$	$C_{X1}-C_{X2}-R_{X1}+R_{X2}$	$C_{Y2}-C_{Y1}+R_{Y1}-R_{Y2}$	$C_{Z2}-C_{Z1}+R_{Z1}-R_{Z2}$
$\left(0,1,0\right)$	$C_{X2}-C_{X1}+R_{X1}-R_{X2}$	$C_{Y1}-C_{Y2}-R_{Y1}+R_{Y2}$	$C_{Z2}-C_{Z1}+R_{Z1}-R_{Z2}$
$\left(0,0,1\right)$	$C_{X2}-C_{X1}+R_{X1}-R_{X2}$	$C_{Y2}-C_{Y1}+R_{Y1}-R_{Y2}$	$C_{Z1}-C_{Z2}-R_{Z1}+R_{Z2}$
$\left(1,1,0\right)$	$C_{X1}-C_{X2}-R_{X1}+R_{X2}$	$C_{Y1}-C_{Y2}-R_{Y1}+R_{Y2}$	$C_{Z2}-C_{Z1}+R_{Z1}-R_{Z2}$
$\left(1,0,1\right)$	$C_{X1}-C_{X2}-R_{X1}+R_{X2}$	$C_{Y2}-C_{Y1}+R_{Y1}-R_{Y2}$	$C_{Z1}-C_{Z2}-R_{Z1}+R_{Z2}$
$\left(0,1,1\right)$	$C_{X2}-C_{X1}+R_{X1}-R_{X2}$	$C_{Y1}-C_{Y2}-R_{Y1}+R_{Y2}$	$C_{Z1}-C_{Z2}-R_{Z1}+R_{Z2}$
$\left(1,1,1\right)$	$C_{X1}-C_{X2}-R_{X1}+R_{X2}$	$C_{Y1}-C_{Y2}-R_{Y1}+R_{Y2}$	$C_{Z1}-C_{Z2}-R_{Z1}+R_{Z2}$

**Table 4:** Simulation parameters.

Parameters	Default Values
Scale of the space	1000 m × 1000 m × 1000 m
Number of nodes	250
Node placement method	Random placement
Range of communication	200 m
Initial energy	100 J
Data packet size	1024 bits
Control package size	128 bits
Transmit power	2 W
Received power	0.2 W

## Data Availability

The raw data supporting the conclusions of this article will be made available by the authors on request.
